# Allergic bronchopulmonary mycosis in *Schizophyllum commune* with positive *Aspergillus*‐specific IgE antibodies: A case report

**DOI:** 10.1002/rcr2.1433

**Published:** 2024-07-16

**Authors:** Hiroshi Takahashi, Masamitsu Hamakawa, Tadashi Ishida, Akira Watanabe

**Affiliations:** ^1^ Department of Respiratory Medicine Kurashiki Central Hospital Okayama Japan; ^2^ Department of Clinical Infectious Diseases Chiba University Research Center for Mycological Medicine Chiba Japan

**Keywords:** allergic bronchopulmonary aspergillosis, allergic bronchopulmonary mycosis, *Aspergillus fumigatus*, *Aspergillus*‐specific IgE antibodies, *Schizophyllum commune*

## Abstract

*Schizophyllum commune* is the third most common causative fungus of allergic bronchopulmonary mycosis(ABPM). Two‐thirds of ABPM caused by *S. commune* can be positive for *Aspergillus fumigatus*‐specific IgE, which can be difficult to diagnose. Our patient presented to our hospital with wet cough for 3 months and chest pain for 3 days. Blood tests showed IgE 1522 IU/mL, eosinophils 688/mm^3^, *A. fumigatus* ‐specific IgE 2.24 UA/mL, and chest computed tomography showed high‐attenuation mucus. Bronchoscopy showed mucus plugs and speculum examination showed filamentous fungi, but various culture tests did not detect *A. fumigatus*, Asp f 1‐specific IgE was negative, and *S. commune* was detected in the culture of bronchial washing. Since he was positive for *S. commune*‐specific IgE and IgG, he diagnosed ABPM caused by *S. commune*. These findings demonstrate the importance of identifying the causative fungus in ABPM by detailed examination.

## Graphical abstract

We report here a case of allergic bronchopulmonary mycosis (ABPM) in which the patient was positive for *Aspergillus fumigatus*‐specific IgE, but after detailed examination, *Schizophyllum commune* was identified as the causative fungus.

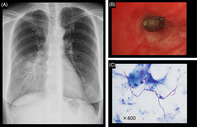

## INTRODUCTION

Allergic bronchopulmonary mycosis (ABPM) is a lung disease caused by a complex allergic reaction to a variety of fungi growing in the lower respiratory tract, with *A. fumigatus* being the causative fungus ABPM is known as allergic bronchopulmonary aspergillosis (ABPA).^1^

*Schizophyllum commune* is the third most common causative fungus of ABPM, followed by *Candida* and *Bipolaris sps*,^1^ and most cases of ABPM caused by *S. commune* have been reported in Japan.^1^ In a recent report, about two‐thirds of ABPM cases caused by *S. commune* were reported to have *A. fumigatus*‐specific IgE.^1^ This fact can sometimes make identification of the causative fungus of ABPM difficult.

We report here a case of ABPM in which the patient was positive for *A. fumigatus*‐specific IgE, but after detailed examination, *S. commune* was identified as the causative fungus.

## CASE REPORT

**FIGURE 1:**
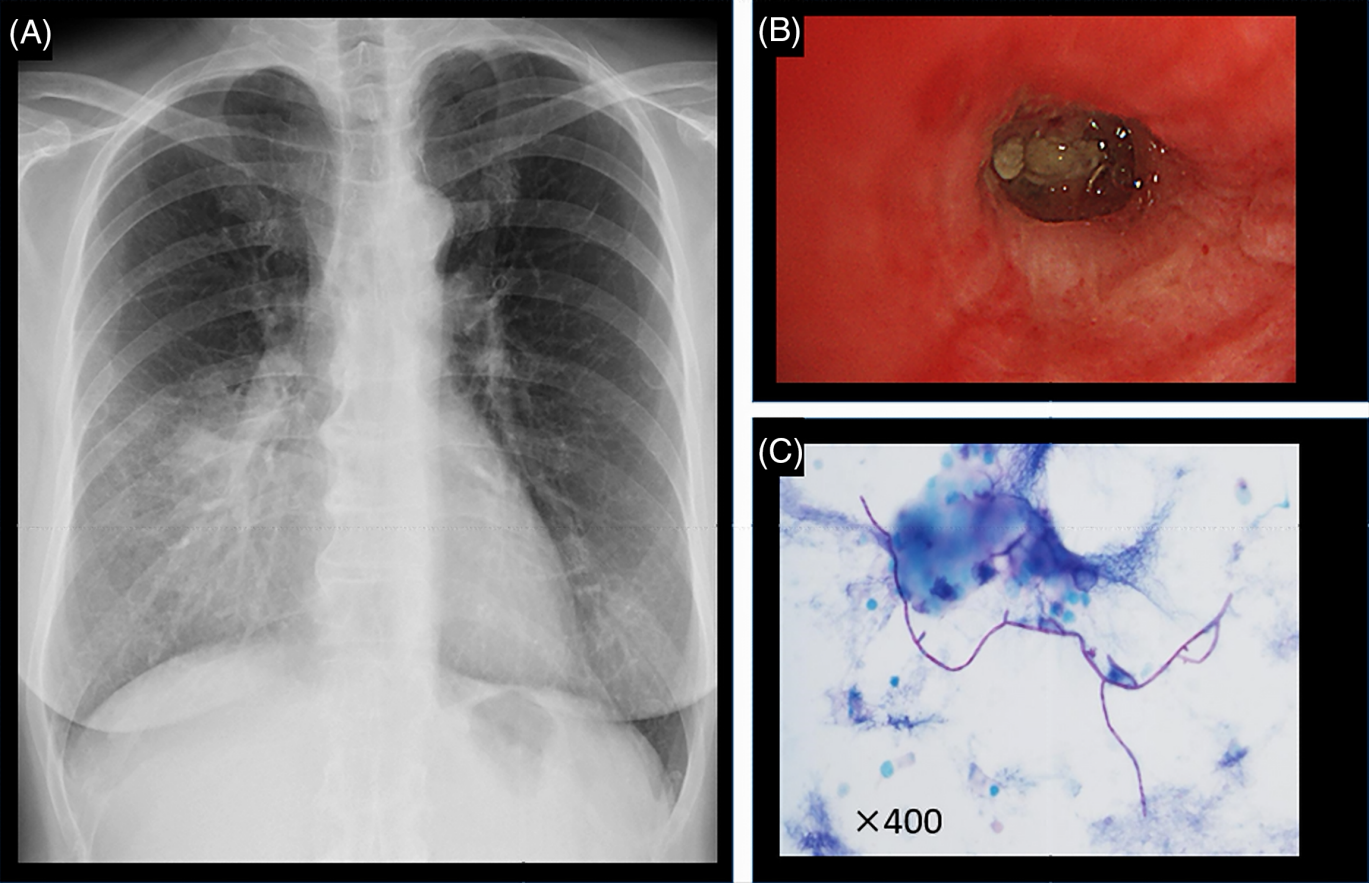
(A) Chest x‐ray shows infiltration shadow in the right lower lung field. (B) Bronchoscopy showed yellow mucus plug in right B^5^ bronchus. (C) Thin club‐shaped filamentous fungi with many undirected branches were detected in bronchial lavage fluid by periodic acid Schiff staining.

**FIGURE 2:**
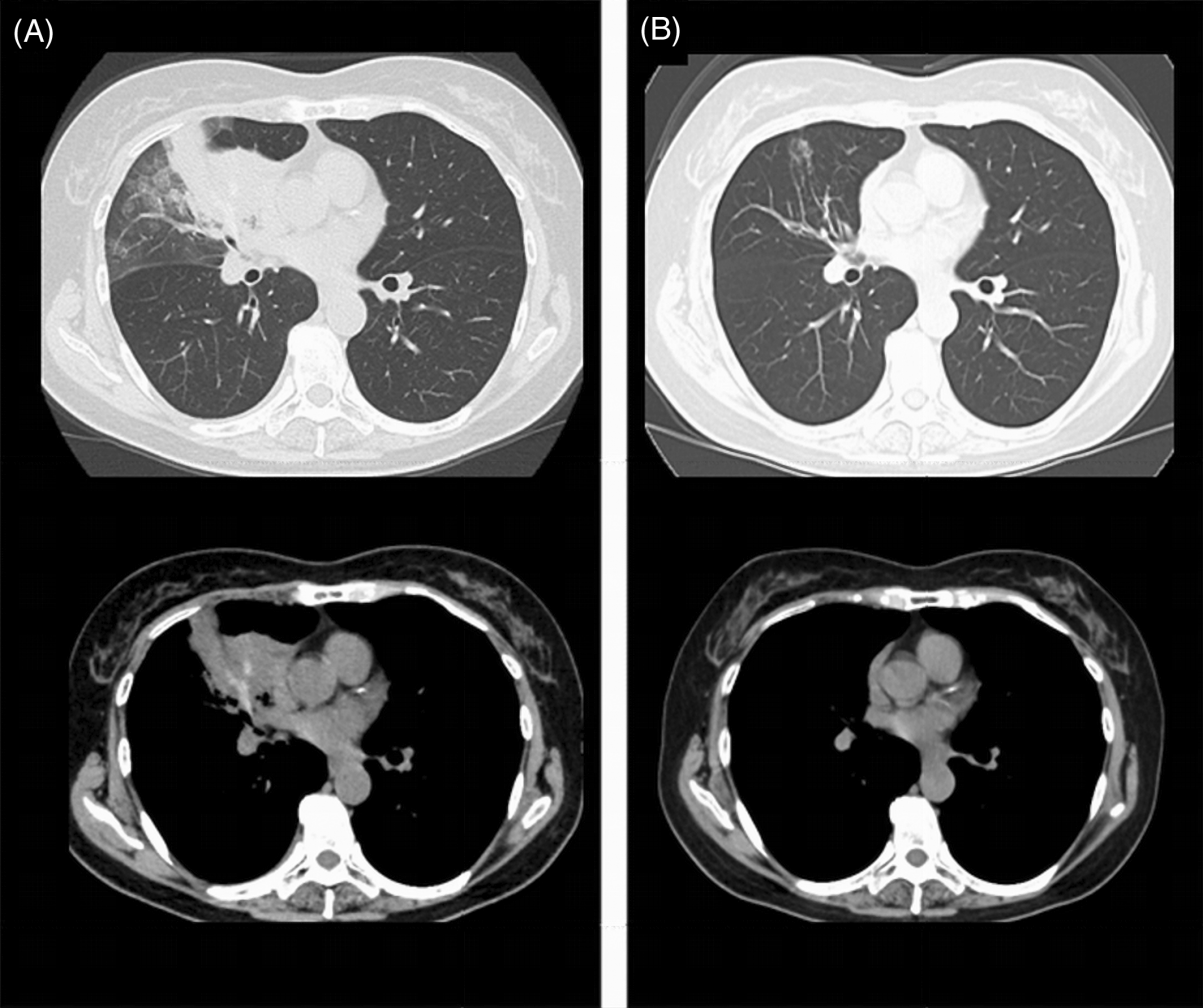
(A) Chest computed tomography showed infiltrative shadow in the right middle lobe and high‐attenuation mucus. (B) At 55 days of treatment, the infiltration shadow and mucous plug in the right middle lobe had resolved.

A 63‐year‐old woman had wet cough for 3 months prior to admission and was aware of chest pain for 3 days prior to admission, so she visited previous hospital. Then, chest x‐ray and chest computed tomography (CT) showed a right middle lobe infiltrating shadow and high‐attenuation mucus (Figures 1A and 2A), so she was referred to our hospital for close examination and was hospitalized. She had no history of bronchial asthma. Her home environment was clean and she had no fungal exposure. She had been a former smoker for 5 cigarettes per day for 13 years.

Vital signs on admission were as follows. Body temperature: 36.7°C, pulse rate: 64 beats/min, blood pressure: 94/64 mmHg, respiratory rate: 16 breaths/min, SpO2: 98% (room air). Auscultation revealed no wheezes or other secondary noises. Blood test findings were as follows: C‐reactive protein 9.64 mg/dL, white blood cell count 8000/mm^3^, eosinophil count 688/mm^3^, IgE 1522 IU/mL, Asp‐specific IgE 2.24 UA/mL, Asp‐specific IgG <1.4 UA/mL. Respiratory function tests were as follows. Forced vital capacity (FVC) 3.49 L (123.3% of predicted), forced expiratory volume in 1 s (FEV1) 2.36 L (109.8% of predicted), FEV1/FVC 67.6%, fractional exhaled nitric oxide test 31 ppb.

We initially suspected ABPA, and bronchoscopy was performed on the fourth day of admission, during which a yellow mucus plug was found in the right B^5^ bronchus (Figure 1B). Although blood tests were negative for Asp f 1‐specific IgE <0.10 UA/mL and *A. fumigatus* was not detected in the bronchial lavage fluid and culture test, thin club‐shaped filamentous fungi with many undirected branches were detected in bronchial lavage fluid by periodic acid Schiff staining (Figure 1C). *S. commune* was cultured on Sabouraud agar medium the bronchial lavage fluid culture. We used potato dextrose agar medium but did not detect *A. fumigatus*. From the above culture results, IgE and IgG antibodies related to *S. commune* were measured using the enzyme‐linked immunosorbent assay method reported by Toyotome et al.^2^ Healthy means for each antibody was calculated by collecting serum from 20 healthy volunteers and averaging the measured results. In the present case, *S. commune* IgG antibody: 1.1260 (healthy mean:0.0891) and *S. commune* IgE antibody: 0.0325 (healthy mean:0.0135) were predominantly elevated. According to the diagnostic criteria reported by Asano et al.^3^ ABPM can be diagnosed when 6 or more of the following 10 criteria are met. (1) history of bronchial asthma or asthma‐like symptoms, (2) peripheral blood eosinophil count (peak) ≥ 500/mm^3^, (3) serum total IgE level (peak) ≥ 417 IU/mL, (4) immediate skin reaction to filamentous fungi or positive specific IgE, (5) positive precipitating antibody to filamentous fungi or positive specific IgG, (6) positive sputum or bronchial washings for filamentous fungi, (7) positive culture of filamentous fungi in mucus plug, (8) central bronchiectasis on chest CT, (9) history of mucus plug exsanguination or central bronchial mucus plug on CT or bronchoscopy, (10) increased density of mucus plug on chest CT. In this case, the diagnosis of ABPM due to *S. commune* was made because more than 8 items were fulfilled except for the absence of a history of bronchial asthma and the absence of central bronchiectasis on chest CT.

**FIGURE 3:**
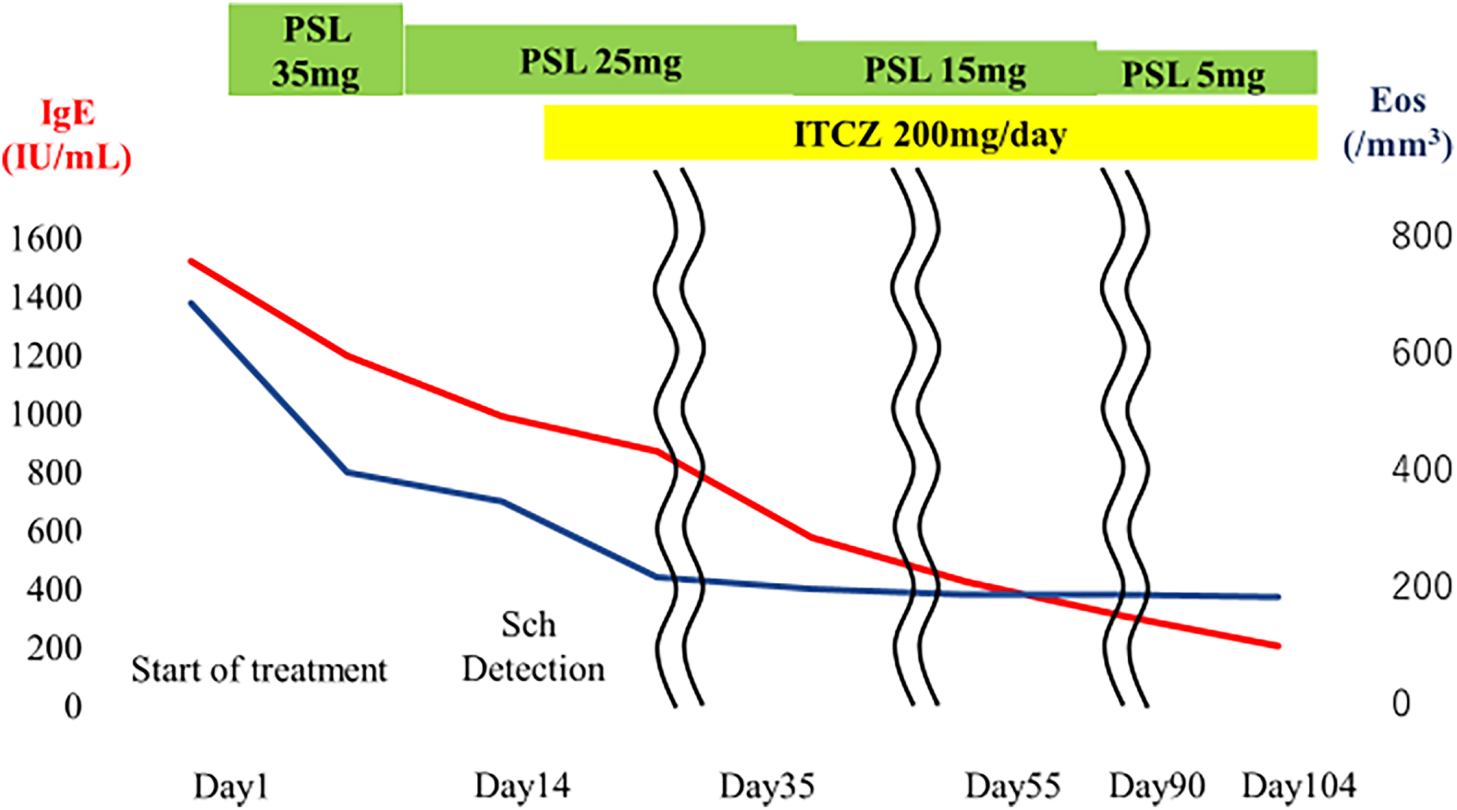
The patient remained free of symptom and imaging relapse until day 104 of treatment, at which time her eosinophil count was 188/μL and IgE was 141 IU/mL. Eos: peripheral blood eosinophil count, IgE: immunoglobulin E, ITCZ: itraconazole, PSL: prednisolone, Sch: *Schizophyllum commune*.

Treatment was started with 0.5 mg/kg of predonisolon (PSL) on the 4th day of hospitalization and the patient was discharged on the 7th day of hospitalization. On the 14th day of treatment, after the diagnosis of ABPM caused by *S. commune*, concomitant use of itraconazole (ITCZ) 200 mg/day was started. Thereafter, prednisolone could be tapered off, IgE tended to decrease over time, and the infiltration shadow and mucous plug seen on chest CT showed a tendency to disappear on day 55 of treatment (Figure 2B). Finally, PSL and ITCZ were gradually reduced, and both drugs were discontinued on day 90 of treatment. The patient remained free of symptom and relapse on imaging until day 104 of treatment, at which time her eosinophil count was 188/μL and IgE was 141 IU/mL (Figure 3).

## DISCUSSION

In this case, *A. fumigatus‐specific* IgE was positive, and we initially assumed ABPA. However, after immunological evaluation based on bronchial washing culture results, we were able to finally diagnose ABPM caused by *S. commune*.

A recent report showed that *A. fumigatus*‐specific IgE and IgG were elevated in approximately 66% and 30% of ABPM caused by *S. commune*, respectively.^1^In this case, *A. fumigatus*‐specific IgE was elevated and *A. fumigatus*‐specific IgG was negative. In fact, several cases of ABPM caused by *S. commune* with positive *A. fumigatus*‐specific IgE have been reported.^4^, ^5^ As in this case, *A. fumigatus*‐specific IgG was negative, and the presence of *A. fumigatus* was not proven in each culture test. Existing reports have indicated the possible involvement of cross‐reactivity between the two fungi as a factor^6^; therefore it is possible that there was cross‐reactivity between the two fungi. The above suggests how difficult it is to identify the causative fungus in the diagnosis of ABPM. Therefore, we believe that comprehensive close examination using culture by bronchoscopy and serological tests is important to identify the causative fungus of ABPM.

In patients with recurrent ABPM, a combination of steroids and antifungal agents has been recommended.^7^ The recurrence rate of ABPA in Japan is as high as 48%,^8^ suggesting that the recurrence rate of ABPM caused by *S. commune* is high at approximately 50%.^9^ Considering the recurrence rate, combination therapy with antifungal drugs and steroids was used in this case. However, compared with ABPA, *S. commune*‐related ABPM may have a milder allergic phenotype, with fewer bronchial asthma complications and lower eosinophil counts and IgE levels,^1^ suggesting that the recurrence rate of non‐Aspergillus ABPM containing *S. commune* remains unclear and needs further verification. The difference in the recurrence rate of ABPM due to the causative fungus may influence the course of future treatment.

Asp f 1 is a specific allergenic component of *A. fumigatus*, and when this specific IgE is detected, it indicates germination of *A. fumigatus* in the bronchus.^10^ When *A. fumigatus*‐specific IgE is positive, Asp f 1 positive is likely to indicate ABPA, while Asp f 1/Aspf2 negative is less likely to indicate ABPA.^10^ Asp f 1‐specific IgG positive has been reported to have a sensitivity of 51.2% and specificity of 84.2% in diagnosing ABPA with a cutoff of 6.6 mg/dL.^11^ When Asp f 1‐specific IgG is positive, it has high specificity when diagnosing ABPA, in addition, *A. fumigatus* co‐sensitization may be considered in ABPM caused by *S. commune*.^11^ In this case, *S. commune* was detected in the bronchial wash culture, and the bronchial wash culture was positive for specific antibodies to *S. commune*, but negative for Asp f 1‐specific IgE only, so we determined that *S. commune* was the causative fungus of ABPM in this case rather than *A. fumigatus*. In this case, the combination therapy with antifungal drugs and steroids was able to be administered early, which is expected to result in a low recurrence rate in the future.

In conclusion, in ABPM, the causative fungus should be identified by detailed scrutiny, as in this case, as the recurrence rate of ABPM may vary depending on the causative fungus.

## AUTHOR CONTRIBUTIONS

Hiroshi Takahashi was responsible for drafting the work, the conception and design of the work, as well as the acquisition, analysis, and interpretation of the data for the work. Masamitsu Hamakawa was responsible for revising the manuscript critically for important intellectual content. Toru Watanabe was responsible for revising the manuscript critically for important intellectual content. Tadashi Ishida was responsible for the final approval of the manuscript version to be published.

## CONFLICT OF INTEREST STATEMENT

None declared.

## ETHICS STATEMENT

The authors declare that appropriate written informed consent was obtained for the publication of this manuscript and accompanying images.

## Data Availability

Research data are not shared.
